# Application of Processing and Packaging Hurdles for Fresh-Cut Fruits and Vegetables Preservation

**DOI:** 10.3390/foods10040830

**Published:** 2021-04-11

**Authors:** Maria C. Giannakourou, Theofania N. Tsironi

**Affiliations:** 1 Laboratory of Chemistry, Analysis & Design of Food Processes, Department of Food Science and Technology, School of Food Sciences, University of West Attica, Agiou Spyridonos, 12243 Athens, Greece; mgian@uniwa.gr; 2 Laboratory of Food Process Engineering, Department of Food Science and Human Nutrition, Agricultural University of Athens, Iera Odos 75, 11855 Athens, Greece

**Keywords:** plant-based foods, hurdles, quality, preservation, processing, packaging

## Abstract

Recently, consumers’ demand for fresh, nutritious, and convenient food has shown a significant rise. This trend has forced increased sales of minimally processed and/or pre-packed fruit- and vegetable-based products. New product development and the diversification of plant-based foods have supported this growth. The food production sector should balance this requirement with the necessity to provide safe food with extended shelf life while meeting consumer demands for novel, nutritious, and affordable food products. The use of alternative “soft hurdles” may result in a decrease in the rate of food deterioration and spoilage attributed to microbial activity or other physiological/chemical degradation reactions. The objective of the article is to provide a systematic review of the preservative effect of the available hurdles implemented during processing and packaging of fresh-cut fruits and vegetables, focusing on recent applications aiming at improving product quality and prolonging their limited shelf life.

## 1. Introduction

Fruits and vegetables play a significant role in healthy human nutrition and possess a high ranking on the list of consumer priorities. The World Health Organization suggested a 400 g daily consumption of fruits and vegetables [1]. In response to this recommendation, several programs relevant to food and health (for example, five-a-day) have been carried out in order to encourage the consumption of fruits and vegetables during the 1990s and later on [2,3]. Despite the indisputable health benefits and convenience, quality preservation, shelf-life extension, and microbial safety assurance of such perishable foods are essentially important. It is well established that postharvest physiological modifications and the potential bacterial contaminations occurring during preharvest and postharvest handling, as well as the additional production steps (e.g., peeling, cutting, slicing, or shredding), cause rapid deterioration and spoilage of fruits and vegetables [4,5]. Several significant foodborne outbreaks have been associated with the consumption of ready-to-eat, fresh, or minimally processed fruits and vegetables, and the numbers of reported cases have shown a constant rise during the past two decades [6].

Several food preservation methods have been applied with the aim to inhibit the growth of microorganisms and preserve the quality of fruits and vegetables. Most processing technologies are based on a single preservation factor (e.g., high temperatures, chill storage, water activity decrease, etc.), which frequently alters the initial quality food profile, especially in terms of sensory, nutritional, and physicochemical properties. However, each one of the preservation techniques presents an optimum to minimum level that influences microorganisms *per se*.

New trends for the production of food products according to consumer demands are minimally or not at all preserved, possessing higher quality, more “natural” sensory properties, produced with or without additives, microbiologically safe and nutritional healthier [7]. The hurdle technology introduces the combined application of conventional and innovative preservation methods, intending to provide a series of preservative factors (hurdles) that microorganisms cannot overcome. Food preservation implies putting microorganisms in a hostile environment to inhibit their growth or shorten their survival or cause their death. The feasible responses of microorganisms to this hostile environment determine whether they may grow or die. In essence, the hurdle approach targets the physiological aspects of microorganisms, and this process involves four major mechanisms: homeostasis, metabolic exhaustion or auto-sterilization, stress reaction, or multitarget preservation of foods [8,9,10]. Aiming to achieve the target of microbial stability, multiple hurdles (appropriate set of applied barriers) are combined to retain the initial superior nutritional and sensory qualities of food. The most important hurdles used in food preservation are the temperature during processing, transportation, and storage (high or low), water activity (a_w_), acidity (pH), redox potential (Eh), preservatives (e.g., natural or synthetic), and competitive microorganisms (e.g., lactic acid bacteria) [8,9]. According to Leistner [8,9], more than 60 potential hurdles have been reported for foods, which may enhance the stability and quality of food products; however, this list is by any means not yet complete. The individual hurdles may be applied concurrently or sequentially, depending on the means of hurdle used and the preservation method of the product [10]. The application of this approach (reported as well as combined processes or barrier technology) has shown its potential as very effective on a wide range of food product types [10,11], as the intelligent combination of hurdles may secure the microbial safety and quality as well as the organoleptic parameters, nutritional value and economic feasibility of a product. This may result in a dramatically different product with unique, new appearance and taste properties. The most important factor for the successful application of hurdle technology is the selection of appropriate hurdles for the target food product. In this context, the purpose of this review article is to summarize the main degradation modes of fresh-cut fruits and vegetables and critically describe the available hurdles implemented during their processing and packaging, focusing on recent applications.

## 2. Principal Modes of Deterioration of Fresh-Cut Fruits and Vegetables

**Figure 1:**
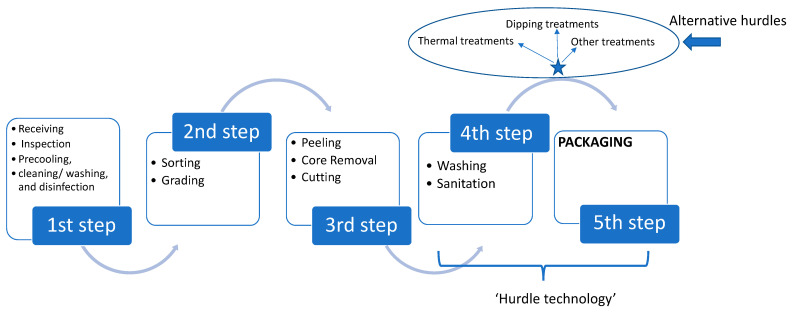
Indicative flow chart of fresh-cut fruits and vegetables’ manufacturing process.

Fruits and vegetables are living food products successively undergoing a ripening and an ageing process, where the plant tissue is actually broken down. The plant-based products undergo various biological processes, which continue long after the harvesting time, such as the respiration process and the ethylene production, which occur at different rates between different fruits and vegetables [12]. The effect of respiration on the quality of fresh produce may include weight loss as a result of oxidative breakdown of substrate molecules—for example, starch, sugars, and organic acids to simpler molecules (e.g., CO_2_ and H_2_O) [13]. As far as fresh-cut fruits and vegetables are concerned, spoilage is often attributed to physiological and microbiological processes and their interactions [14]. An indicative flow chart of such products manufacture, including a variety of treatments prior to the application of “hurdle technology,” is shown in Figure 1 [15].

In this section, the most important quality characteristics and the main deterioration modes of these perishable plant tissues will be briefly outlined, with the purpose to better demonstrate the necessity of specific barriers/packaging approaches for their preservation. It is not in the scope of this review article to describe the modes of deterioration of such perishable tissues in detail, an issue that is explicitly presented in [16,17,18].

### 2.1. Microbial Spoilage

As detailed in [16,17], since fruits and vegetables constitute good sources of nutrients and water, a variety of microorganisms could potentially grow under ambient conditions. A microbial attack can take place either in the field or during postharvest and processing operations. Spoilage bacteria include species such as Pseudomonadaceae, Enterobacteriaceae, and other species within lactic acid bacteria (mainly *Leuconostoc mesenteroides*). Depending on the composition of the plant matrix, many different yeast and mold species have been identified in fresh-cut fruits and vegetables. In most cases, however, the presence and proliferation of these microorganisms affect severely product quality but do not actually jeopardize consumers’ health. Focusing on pathogens, only a few species have been found in fresh-cut produce, such as *Escherichia coli* O157:H7, *Salmonella* spp., *Shigella* spp., Listeria monocytogenes, Campylobacter, and certain viruses and parasites. There are numerous published investigations that have explicitly described the effect of several treatments on the microbiological decay of a variety of horticultural products, e.g., in carrot [19], cantaloupe [20,21,22], apple wedges [23], strawberries [24], etc.

### 2.2. Physiological Activity

Fresh-cut produce requires wounding the plant tissue, which frequently initiates a sequence of respiratory, metabolic, and enzymatic activities that result in important quality degradation [18]: texture deterioration, enhanced ripening and senescence, formation of unpleasant off-flavors, degradation of color, and other undesirable changes. All these reactions occurring in fresh-cut fruit and vegetable products decrease product shelf life significantly and, consequently, can render the product unmarketable. Postharvest behavior of climacteric tissues is characterized by increased respiration, accelerated ethylene-a gaseous ripening plant hormone-production, and several other composite metabolic activities that may trigger ripening and senescence of such vulnerable plant products [18,25]. The release of numerous quality-degrading enzymes, such as polyphenol oxidases, cellulases, pectolytic enzymes, amylases, and peroxidases, causes serious sensory changes, such as discoloration, softening, the production of off-flavors and off-odors, etc. [26,27]. Another aspect of the dynamic behavior of such products is related to water loss, widely known as “transpiration.” Fresh-cut plant tissues are reported to lose water to a higher extent than intact products, which is attributed to an increased surface-area-to-volume ratio resulting from cutting procedures during minimal processing. On the other hand, a chilled storage temperature [28,29] and atmosphere composition may also cause several disorders since they affect both respiration rate and the nutritional quality of fresh-cut produce. Nevertheless, it is worth noticing that the fresh-cut product does not suffer from chilling injury, a disorder that is frequently observed in the intact counterpart [17]. Taking into consideration that all of these physiological pathways downgrade product sensory and nutritional quality, the challenge of the current research lies on testing potential hurdles in order to counteract this physiological decay, including low temperatures, edible coatings, antioxidants, acidulants, antimicrobial agents, ethylene inhibitors, MAP packaging, etc. In recent literature, there are several interesting studies on quality degradation of minimally processed fruits and vegetables due to physiological activity, namely for pomegranate [30], celery [31], broccoli [32], tomato [14], asparagus [33].

### 2.3. Factors Related to Degradation of Sensory Characteristics

#### 2.3.1. Water Loss

Moisture loss as a result of transpiration during storage and transportation is a significant factor that affects the quality and marketability of sensitive products, such as fruits and vegetables. During postharvest handling, fresh fruits and vegetables tend to lose moisture through their skins, stomata, cuticle, and other structural components, especially when the surrounding atmosphere has low relative humidity. Transpiration phenomena are caused by a difference in water vapor pressure between the product surface and the environment [18,26]. It is worth mentioning that the evaporation occurring at the product surface is an endothermic process, which will decrease product temperature. Water loss depends on intrinsic factors, such as surface morphology, size, surface-to-weight and volume ratio, maturity stage, physical injuries, as well as extrinsic factors, such as temperature, relative humidity, and airflow around the product. As readily observed [26,34], some plant tissues are more prone to water loss, such as leafy vegetables, e.g., lettuce, which wilt and shrivel rapidly, whereas others, like apples and pears, are more resistant to water loss. Fresh-cut products are more susceptible to quality degradation than whole products due to the increased rates of transpiration caused by their increased surface-to-volume ratio. This water loss process has serious adverse effects on the quality attributes of such perishable commodities, including loss of turgidity, shriveling, wilting, and withering [35]. There are numerous studies measuring these indices in order to assess product degradation [19,30,36,37,38,39].

#### 2.3.2. Texture Changes

The texture attributes of plant tissues vary during postharvest handling, as they depend on many factors, such as the stage of maturity, water stress, storage temperature and relative humidity, rough handling, and the ripening process. Changes observed in several products include softening, turgor loss, and increased elasticity or toughness, leading to a significant product quality deterioration. These negative phenomena may be caused due to the transpiration process or the activity of several enzymes or mechanical injuries that occur during transport and storage. Enzymes, such as β-galactosidase, polygalacturonase, pectin methyl esterase, cellulose, phenylalanine ammonia-lyase, peroxidase, and cellulase, may lead to cell-wall modification and significant pectin degradation. Examples of such textural defects involve pectin degradation in strawberries [40] and tomatoes [39,40], toughness development in asparagus [33], loss of crispness in lettuce and spinach [41], hardness increase in carrot [19,42], firmness decrease in celery [38], mushiness in cantaloupe [43], changes in broccoli [32], etc. [44].

#### 2.3.3. Color Changes

Color is considered one of the most decisive factors for a consumer’s acceptance or rejection of commodities, such as fresh-cut fruits and vegetables, and is frequently used as an indicator of the overall quality level and maturity stage of the final product [17]. Color is significantly affected by factors, such as the particular cultivar, temperature and relative humidity conditions, and postharvest handling procedures. Color changes are induced by anabolic or catabolic reactions of pigment compounds, including chlorophylls (green), anthocyanins (red, blue, and purple), and carotenoids and flavonoids (yellow and orange) [45]. These changes may occur as a consequence of the ripening process, but they also can be induced by mechanical injuries on cell tissues during handling and fresh-cut processing. Another frequent cause of color degradation in fresh-cut fruits is associated with enzymatic activity (for example, of PPO (polyphenol oxidase) and POD (peroxidase)) that follows cell-wall damage, which enables the immediate contact of enzymes, substrates, and oxygen. The browning of apples, peaches, pears, avocados, etc., is considered a major defect of these commodities and depends on the presence of phenolic compounds (the necessary substrate), the activity of polyphenol oxidase (PPO), and the concentration of antioxidants within the fruit cells [46]. Bhatia et al. [30], Zhao et al. [36], Massolo et al. [31], Paulsen et al. [32], Kumar et al. [39], Sucheta et al. [40], and Lwin et al. [33] have studied the color changes of minimally processed fresh-cut produce as an index to describe product degradation.

#### 2.3.4. Flavor/Taste and Nutritional Changes (Changes of Composition)

It is well known that the plant tissue’s composition is constantly changing, not only during growth and ripening but also after harvest, leading to either positive or negative quality attributes [16]. Among those compositional modifications, the soluble solids content and acidity (and especially their ratio) is frequently used as a quality criterion for product selection for processing and time of harvest. Such changes are more pronounced for climacteric commodities since they continue to ripen even after harvest. Flavor characteristics of most fruits are greatly influenced by their sugar concentration (sweetness), organic acids (acidity), phenolic compounds (astringency), and specific volatiles (aroma) [45]. Volatile losses can be associated with the ripening process, unsuitable storage conditions, or enzymatic activity of the corresponding enzymes, such as peroxidases and lipoxygenases. As far as nutritional factors are concerned, fresh fruits and vegetables are considered good sources of vitamins, minerals, dietary fiber, and several types of compounds possessing an antioxidant activity (flavonoids, carotenoids, polyphenols, and other phytonutrients). A significant loss of such substances is measured during handling and processing and after harvest. These losses are mainly caused by physical damage of the cell wall, inappropriate conditions of temperature and relative humidity during storage, and chilling injury of sensitive commodities [45]. In Massolo et al. [31], Sucheta et al. [40], Zou et al. [22], Rodríguez-Arzuaga et al. [23], and Avalos-Llano et al. [24] sensory changes of selected fresh produce have been extensively studied.

## 3. Hurdles Applied in the Preservation and Shelf-Life Extension of Fresh-Cut Fruits and Vegetables

As frequently observed in relative surveys, consumers recently tend to prefer perishable foods with minimum but adequate processing, so the nutritional/sensory profile of the processed food will not be substantially altered. In this work, the focus is drawn on fresh-cut fruits and vegetables; minimally processed produce is reported as “the fruits and vegetables washed, physically processed (peeling, cutting, slicing or shredding), packaged and maintained with refrigeration” [6,47]. Being still-respiring matrices, the main difference from their raw counterparts is that the respiration process is increased by processes, such as cutting, slicing, low-temperature heat treatments, and preservatives [48]. Furthermore, they do not resemble dehydrated foods, especially due to their texture attributes and water activity (a_w_), which is higher than 0.95. On the other hand, dehydrated plant tissues are handled as shelf-stable at ambient temperatures, and thus cold-chain management is not applied. As far as comparison with thermally processed foods is concerned, minimally processed fruits and vegetables cannot be considered as “commercially sterile.”

Different types of hurdles can be used in fruit and vegetable processing, including thermal treatments, low-temperature storage, regulation of acidity, water activity decrease, the use of the appropriate preservatives, etc. [8,9,10]. In all cases, the selection and sequence of hurdles depend on the type of microorganisms, and the main purpose is to satisfy the safety standards without compromising the food quality and consumer health [49]. The careful and well-documented choice of hurdles, in addition to the intensity of each, and the sequence applied to obtain a specific safety and quality target, are expected to show significant potential for the future of minimally processed fruits and vegetables [50].

Regarding fresh-cut fruits and vegetables, as already discussed, the prevailing factors causing irreversible spoilage and organoleptic rejection are related to the potential microbial and enzymatic activity; thus, all potential preservation techniques, the so-called “hurdles,” aim at an effective microbe and enzyme inhibition [48]. To obtain the desired shelf-life improvement, some traditional methods, such as heat preservation (preferring mild heat treatments), use of chemicals (e.g., acidulants, antioxidants, chlorine, antimicrobials, sanitizers, etc.), low-temperature preservation, application of proper irradiation, oxidation/reduction (O/R), decrease of water activity (a_w_), and appropriate packaging (e.g., Modified Atmosphere Packaging, MAP, edible coatings, etc.), can be effectively applied. The combined application of the aforementioned technologies, considering the synergistic effect of the different preservation hurdles or barriers, may be selected. These barriers take into consideration intact or destroyed enzyme systems in the living tissues, mainly polyphenol oxidase (PPO), peroxidase (PO), or pectinases, polygalacturonase (PG) and pectinesterase (PE), and other respiratory-related enzymes.

Regarding fresh-cut fruits and vegetables, the washing step and the sanitation procedure play a significant role in the final food safety since these commodities are considered as “ready-to-eat” foods. The purpose is to remove pesticide residues, dirt and foreign objects, and microorganisms responsible for quality degradation and spoilage [51,52]. Besides the use of chemicals for disinfection, other physical technologies, such as heat treatments, UV-treatment, high pressure, pulsed electric field, pulsed light, oscillating magnetic fields, low-dose gamma irradiation, and ultrasound treatments, have been investigated to reduce or eliminate microorganisms, usually with more than one technique in an appropriate combination [52,53]. In this section, the main hurdles applied in fresh-cut fruits and vegetables are briefly described, and a Table (Table 1) including indicative studies is presented.

### 3.1. Conventional Hurdles

#### 3.1.1. Short Time Heat Treatments

This is one of the oldest and most popular forms of preservation, which aims at reducing microorganisms and inhibiting enzyme activity in plant tissues (the well-known blanching step). The major problem in such perishable products is that heat is associated with significant degradation of flavor, texture, color, and nutritional quality. The main treatments applied in fresh-cut fruits and vegetables include short-time hot water treatments [77,78,79,80,81] that aim to control the fresh produce surface microflora. Other heat treatments include hot water rinsing and brushing (HWRB) [82], mild Heat Shock [50,83], hot water blanching [42,84], etc. As discussed in Sivakumar and Fallik [82], short-time heat treatments are becoming more attractive in the fresh-cut produce industry as an effective means of preventing the negative effects of enzymatic browning and an alternative to chemical preservation.

#### 3.1.2. Low Temperatures

Chilled storage after treatment is a necessary step in fresh-cut fruits and vegetables, aiming at slowing down microbial growth and inhibiting extensive enzyme activity. Pre-cooling is a common treatment step frequently applied by fresh horticultural produce industries due to its low cost, simplicity, and convenience [33,85].

#### 3.1.3. Chemical Preservation (with or without pH Control)

This category of hurdles is very important for fresh-cut fruits and minimally processed plant tissues because it includes agents used for washing and sanitizing purposes [50]. The food industry relies on wash-water sanitizers to decrease initial bacterial populations immediately after cutting and as a tool to preserve their quality and extend their shelf life [51]. Some of the common chemicals include chlorine-based compounds (hypochlorite, chlorine dioxide, acidified sodium chlorite, electrolyzed oxidizing water, etc.), organic acid formulations, hydrogen peroxide (H_2_O_2_), ozonated water and gaseous ozone (O_3_), alkaline products, and iodine detergent products. In recent literature, there are numerous investigations reporting the effective use of chemical compounds for sanitizing fresh-cut produce [86,87,88,89,90].

Besides washing and sanitizing agents, other chemical compounds have been extensively applied to inhibit microbial spoilage and preserve the quality of low-acid vegetable-based products, acidified low-acid products, and high-acid fruit products. Such substances include antimicrobials, such as organic acids, medium-chain fatty acids, fatty acid esters of polyhydric acids, sugar and salt, L-ascorbic acid, and EDTA [91]; those preservatives that act as antioxidants are widely used to prevent enzymatic browning, inhibit pigment discoloration, and protect against the loss of sensory and nutritional attributes. In Gurtler et al. [92], a review is presented on cases where antimicrobial compounds and mild thermal processing have been used in combination to enhance the inactivation of foodborne pathogen populations in perishable foods, including cut lettuce, cut or shredded cabbage, shredded carrot, and baby spinach leaves.

#### 3.1.4. Water Activity Decrease

Water activity reduction can be a very effective way to limit microbial and enzyme activity because the majority of fresh-cut fruits and vegetables have an a_w_ ≥ 0.98. This method involves either the removal of a certain amount of moisture from the foods (a type of dehydration) or the simultaneous impregnation in a medium or high osmotic pressure (osmotic treatment). It is obvious that the reduction of water activity cannot be used as the sole “hurdle” since that would lead to extensive dehydration of the matrix with unpleasant effects on the nutritional and sensory attributes of the raw tissue. As will be shown through specific examples, the use of a_w_ reduction in combination with another preservation method can be an effective way to retain raw material superior quality, extending its shelf life at the same time. This hurdle is implemented in Dermesonlouoglou et al. [55] and Medina et al. [93].

#### 3.1.5. Biological Methods (Biopreservation)

This process is mainly based on the use of natural or controlled microflora, such as lactic acid bacteria and/or metabolites, to improve the shelf life and the safety of food products [94]. This species is known to produce numerous antimicrobial metabolic compounds, such as bacteriocins, diacetyl, hydrogen peroxide, and organic acids, which may show a detrimental effect on pathogenic microorganisms [95,96].

### 3.2. Emerging Techniques in the Hurdle Integrated Technology

Recent developments in hurdle technology involve the introduction of innovative techniques as part of the hurdle sequence [97,98], for example HHP [55,99,100], PEF [101,102], UV-C (short-wave ultraviolet) [100,103,104], ozone [105], pulsed light [106], High Power Ultrasound [100], natural antioxidants extracted from food waste, etc. In Alzamora et al. [97], an overview of the main emerging “nonthermal” factors is presented, together with their mode of action, potential advantages and disadvantages, and the combined processes (complimentary hurdles), which have been used for fruit preservation. In another interesting recent publication, the most important innovative developments applied to fresh-cut fruits are presented, including physical, chemical, and biopreservation methods. High Hydrostatic Pressure (HHP) is a technique that is based on the instantaneous and uniform application of pressure around 100–800 MPa, below 0–100 °C, from seconds to about 20 min, aiming at membrane disruption, protein denaturation, leakage of vegetative cell content, and dissociation of ribosomes. Short-wave ultraviolet light (UV-C) is radiation from the 200–280 nm region of the electromagnetic spectrum. This technique is well known to cause extensive damage to DNA, membrane integrity, and enzyme activity. Regarding fresh-cut fruits or minimally processed vegetables, UV-C disinfection is a common procedure in the postharvest treatment for the surface inactivation of microorganisms [53], followed by other hurdles, such as MAP or cold storage, for a further shelf-life extension. Pulsed Electric Fields (PEF) involves the application of short electrical pulses of high voltage (5–50 kV/cm, pulse duration few μs) for microbial inactivation while retaining the plant food superior quality. The underlying mechanism is the disruption of the cell membrane and the loss of membrane permeability. The high-power ultrasound (US) technique is based on energy generation by sound waves (5 W/cm^2^; 20–100 kHz), and it is well known to cause extensive cellular damage and cell lysis attributed to cavitation. Another emerging hurdle is pulsed light (PL), which is based on high-intensity light pulses of short durations within a wide wavelength range, from ultraviolet to the near-infrared region (200–1100 nm). Besides the significant microbial reduction obtained in a very short treatment time due to DNA and cellular components damage, its low environmental impact and high flexibility are the main benefits of the PL technique [106]. Cold plasma is another nonthermal processing technology that aims at reducing microbial populations on produce surfaces as an alternative to chemical disinfecting agents [100,107,108]. Recently, scientific research has investigated the potential of using dense phase carbon dioxide (DP-CO_2_) as an alternative nonthermal treatment to inactivate microorganisms and enzymes in minimally processed fruits and vegetables [108,109,110,111]. Another interesting “hurdle” involves the use of natural compounds with antioxidant capacity as an effective substitute for synthetic additives. Additionally, the benefits of such an approach are maximized when those bioactive compounds are extracted from food industry byproducts, as a part of sustainable exploitation of food waste. In Venturi et al. [112], high-added-value antioxidant compounds were extracted from organic potato byproducts and were effectively applied in the pretreatment of cubes of fresh-cut apple, replacing other potentially hazardous chemicals successfully. Similar positive findings regarding the benefits of exploiting food byproducts for extending the short shelf life of several cut horticultural products are presented in [113,114].

The preservation using irradiation includes infrared heating, microwave, UV-light, ionizing irradiation, etc. Electron Beam Irradiation (EBM), a method that does not require radioactive isotopes to generate ionizing radiation, has been shown to cause significant reduction of pathogenic microorganisms and has the potential to increase the shelf life and maintain the freshness of fresh-cut fruits [95,115,116,117].

**Figure 2:**
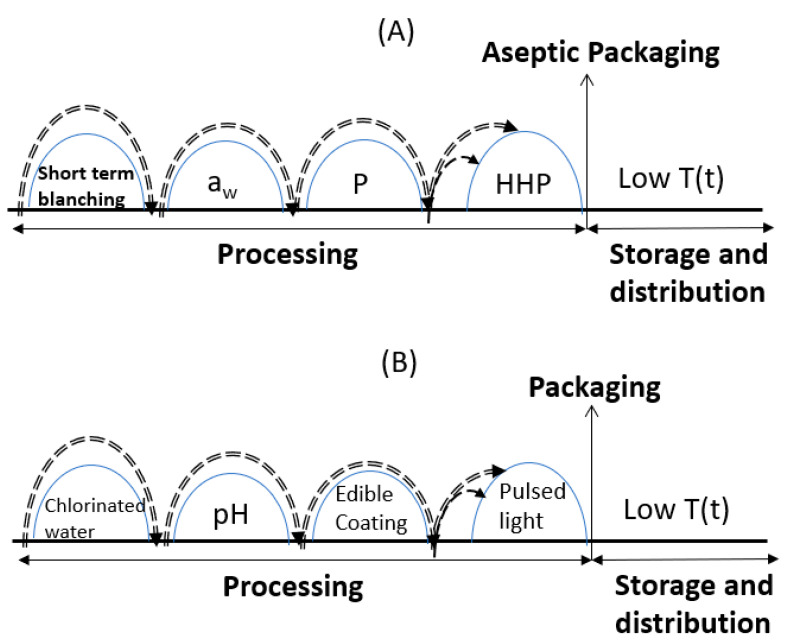
Indicative examples of the hurdle concept on the preservation of fruits, based on (**A**) Dermesonlouoglou et al. [59] and (**B**) Moreira et al. [56].

An illustrative presentation of the hurdle concept application is presented in Figure 2, for some indicative cases of Table 1. In Figure 2A, a fresh-cut product (peaches and apricots) receives a short heat treatment (blanching) in order to reduce the level of remaining enzymes and decrease the initial microbial load present in the raw material. The second hurdle involves the simultaneous application of an a_w_-lowering process, namely the osmotic dehydration of cut pieces, immersed in a solution containing a preservative agent (a natural antimicrobial product-signaled as P), which directly affects the microbial systems. The High Hydrostatic Pressure (HHP) step aims at both enzyme inhibition and an additional microbial inactivation (cold pasteurization). This product would be packaged and retained at low temperatures throughout its shelf life during storage, distribution, and marketing (data obtained from Dermesonlouoglou et al. [59]). In Figure 2B, fresh-cut apples are first washed with chlorinated water (slight microbial reduction) and then dipped into an ascorbic acid/calcium chloride solution, lowering the pH and causing an antibrowning effect (through enzyme inactivation). The next hurdle involves an edible-coating treatment, which reduces respiration, water loss, and oxidation reaction rates, which slightly affects both enzyme and microbial inactivation. Finally, a pulsed-light treatment is conducted, aiming at a further microbial reduction. This product would be packaged and retained at low temperatures throughout its shelf life during storage, distribution, and marketing (data obtained from Moreira et al. [56]).

## 4. Protection of Fruits and Vegetables by Appropriate Packaging Methods

The major concern in purchasing ready-to-eat fresh-cut fruits and vegetable products refers to their short shelf life as a result of the rapid quality deterioration at the postharvest stages, which results in undesirable appearance and decreased palatability. For example, pre-packed fresh-cut leafy salads are significantly perishable, and their shelf life under refrigeration (≤5 °C) ranges between 7 and 10 days. The shelf life of fruits and vegetables can be calculated based on bacterial and chemical modifications. The complex indigenous spoilage microflora comprises *Pseudomonas* spp., lactic acid bacteria, *Enterobacteriaceae* spp., yeasts, and molds. Additional factors affecting the quality may be browning and enzymatic softening, which may be partially be attributed to enzymes from microorganisms [66,118,119].

The use of appropriate packaging is necessary for the minimization of physical damage of fruits and vegetables and to obtain an optimal shelf life. In some cases, selected types of packaging (for example, healthy, fun, plain, etc.) of fruits and vegetable products may be selected in order to influence specific target groups’ (e.g., children) health and taste evaluation. According to Dial et al. [120], children have been influenced by some aspects of packaging, rating healthy and fun packaging similarly. In general, the most commonly used method of packaging for fresh produce is the application of a fiberboard carton; however, in most cases, an additional internal packaging layer is appropriate in order to limit damage from abrasion. For this purpose, tissue paper wraps, trays, cups, or pads may be used. In the case of very delicate fruits, small packs with relatively few layers of fruits are applied for the reduction of compression and additional protection from damage. Fruits may also be wrapped individually using tissue or waxed paper. This technique may reduce the contamination with disease organisms within a package [12].

Smart packaging, considering active packaging methods and quality management tools, such as gas and moisture control, antimicrobial and/or antioxidant packaging systems, smart labels, and edible films or coatings, may be considered as novel packaging methods, which have been reported to result in improved quality and prolonged shelf life of sensitive food products.

### 4.1. Gas and Controlled/Modified Atmosphere, Edible Coatings and Other Forms of Packaging (This Will Be Described in Detail in the Following Section)

Modified atmosphere packaging (MAP) is a method of food preservation which has been originally designed for the packaging of fresh produce. Alternative polymeric films have been applied to pack fresh products for over forty years. Several of benefits have been reported in literature, including limitation of water loss, protection from skin abrasion and inhibition of microbial contamination during production and storage. A barrier to the spread of decay from one unit to another has been also provided [46]. Packaging films may also prevent the flow of respiratory gases, based on the relative permeability of the applied packaging films. This may result in the development of lowered O_2_ and higher CO_2_ concentrations in the package headspace and, as with control atmosphere storage (CAS), this may result in reduction of the respiration rate of the product and thus potentially prolong shelf life [12].

MAP may be considered as an active or passive dynamic packaging system, aiming at modifying the heaqdspace gas concentration within the food package. The passive approach relies on the application of the natural initial gaseous composition and the interaction between the product respiration rate gas permeability through the packaging. In the active approach of MAP, gases of required composition are flushed into the food packaging so as to achieve a rapid equilibrium atmosphere [13]. Improper control of respiration may result to undesirable phenomena from low oxygen levels to anaerobic respiration, accelerated physiological decay and limited shelf life [121]. Appropriate design of modified atmosphere and humidity packaging of fresh fruits and vegetable products has been the main objective of several research studies. Tsironi et al. [66] developed and validated the applicability of an Arrhenius type model for shelf-life prediction of ready-to-eat fresh cut salads (lollo rosso lettuce, lollo verde lettuce and rocket), in realistic distribution temperature conditions in the food supply chain. The quality level and shelf life calculated after 2–3 days of simulated domestic storage (corresponding to the time of consumption) ranged 1–8 days at 4 °C and was predicted within acceptable statistical variations by the validated kinetic models.

Dermesonlouoglou et al. [67] developed shelf-life models for dandelion, that allowed for clalculations of the quality level and shelf life under alternative packaging types and storage temperatures. Microbial growth and modifications of different quality indices of dandelion packed in air-perforated films and under modified atmospheres were evaluated under different storage conditions. Jalali et al. [13] proposed a mathematical model for the prediction of the dependent variables of packaging design, as for example gas composition in the headspace, humidity and water vapour condensation dynamics under different environmental conditions, resembling the real supply chain for fresh fruits and vegetable products (such as plum and strawberry), based on specific properties of fresh food products and packaging materials. Recently, advanced techniques, as for example equilibrium MAP for dynamic modification of the headspace atmosphere have been applied for the extension of shelf life of high value fresh produce [122]. Successful applications of equilibrium modified atmosphere packaging include a wide variety of fresh plant-based products, such as broccoli, cauliflower and carrots [123], peach and cherry tomatoes [68]. A main disadvantage of this method is the extensive use of plastic materials, which may result in large amounts of plastic waste. At the same time, this method of packaging requires low temperature storage, i.e., 0–5 °C, in order to maximize the shelf life of the packaged products. As the storage temperature increases, O_2_ requirements from tissues increase as well, while tolerance to CO_2_ decreases [68].

### 4.2. Active Packaging of Fruits and Vegetables (Including Antimicrobial Packaging)

In addition to the appropriate function for protection and assurance of the safety and integrity of the foods, recently, the application of packaging targets complementary functionalities. Smart packaging can contribute to shelf-life extension and may provide crucial information about the safety and quality level of food; and thus, it can enable the efficient management of the cold chain, minimization of food waste, and enhancement of consumer protection and public health. The “smartness” of packaging is based on the possibility to communicate critical information regarding food quality, such as package integrity and the time-temperature history of the packaged food product. Smart packaging may also provide direct information to the users about the quality level of the particular food, such as the freshness indicators that may provide information regarding the freshness and quality of the packaged food [124,125,126].

According to the EU Guidance to the Commission Regulation (EC) No 450/2009, the packaging is considered as active in case that it provides functions beyond the conventional protection and inert barrier to the external environment [127]. The main difference between intelligent and active packaging is that active packaging involves appropriate detection and response to modifications of the internal or external environment in order to adjust the properties of the package. Intelligent packaging turns on and off based on modifications of the external or internal environmental factors and communicates information to the consumer regarding the quality level of the packaged food [128,129]. Intelligent materials are defined on the basis of EC/450/2009 as tools that track the status of packaged food or its surrounding environment.

Active packaging includes different aspects, such as physiological processes (for example, fresh fruit and vegetable respiration), chemical modifications (for example, lipid oxidation), physical changes (for example, loss of moisture), and microbial activity (e.g., microbial), which may influence the shelf life of packaged fruits and vegetables. Through the implementation of suitable active packaging systems, these conditions can be handled in several different ways and in turn, the quality deterioration may be significantly delayed depending on the requirements of the packaged product. The specific functionality of active packaging depends on the active compounds that are incorporated into the packaging system. For instance, an antimicrobial active packaging system is based on the incorporation of antimicrobial compounds into the packaging material with the aim to delay or prevent microbial growth or contamination at all stages from transportation to the final consumption [6].

The presence of oxygen in fresh food packages may have a detrimental effect on the quality parameters and sensory attributes. It may cause deteriorative reactions, such as loss of nutrients, color changes, off-flavor development, and microbial activity. It has also a significant effect on the respiration rate and ethylene production in fruits and vegetable products. The elimination or even exclusion of O_2_ has been an aspect of significant effort in the preservation of food products. Passive barrier packaging materials, as for example, high barrier materials and multilayer systems, which contain ethylene-vinyl-alcohol co-polymers or aluminum foil, may be used for the packaging of O_2_ sensitive foods [130], as well as high barrier nanocomposites [131]. However, the absolute elimination of O_2_ into the headspace or the dissolution of O_2_ on the food surface or O_2_ permeated into the package wall cannot be attained by passive methods. Therefore, O_2_ scavengers, also referred to as O_2_ absorbers, can be advantageous to retain food quality by decreasing food metabolism, reducing oxidative rancidity, inhibiting undesirable oxidation of labile pigments and vitamins, delaying enzymatic browning, controlling enzymatic discoloration, and inhibiting the growth of aerobic microorganisms [132,133,134]. O_2_ scavengers have been reported as the most commercially important type of active food packaging. O_2_ scavengers are based on compounds, such as powdered iron or ascorbic acid, the former being more widely applied. In absorbers, the absorption capacity and rate are constant, representing their two typical and primary parameters. Despite the well-documented absorption potential of commercial sachets, the absorption rate, a parameter of fundamental importance for food quality, has been evaluated in limited studies. The most commonly used O_2_ scavengers are small sachets containing various iron-based powders along with a series of catalysts that scavenge O_2_ within the food package and irreversibly transform it into a stable oxide [135]. Oxygen scavengers may be applied alone or combined with MAP. In the case that they are applied alone, MAP machinery will not be needed, and the packaging stage will be accelerated. However, the main amount of ambient O_2_ is removed by MAP in standard commercial practice, and the residual oxygen left inside the package is removed by a relatively small and inexpensive scavenger [136]. The Ageless^®^ O_2_ absorber was introduced in 1977 by Mitsubishi Gas Company. This system consisted of reduced iron salts, activated by H_2_O and placed in a sealed gas-barrier package, where Fe oxidized to a ferric state. The reduced iron-containing sachets were applied with appropriate O_2_ scavengers to enable the preservation of the contained foods in large containers. Later on, Toppan Printing (Tokyo, Japan) launched an ascorbic-acid-based O_2_ scavenger. During the nineties, Toyo Seikan Kaisha Ltd., Tokyo, Japan (with Oxyguard^®^), W.R. Grace and Company, Columbia, MD, USA (with Daraform^®^ 6490 and Cryovac^®^ OS1000), and Multisorb Technologies Inc., New York, NY, USA (with FreshMax^®^) provided the most updated O_2_ scavengers relevant with the current O_2_ absorber systems, such as OxySorbTM (Wholesale Group International Pty. Ltd., Australia) [137]. Charles et al. [138] reported that the application of O_2_ absorbers on packaging may extend the shelf life of tomatoes. By the application of a low-density polyethylene film in conjunction with an iron-based O_2_ absorber, the CO_2_ peak was suppressed. Additionally, oxygen absorbers have been applied for the inhibition of β-carotene oxidation in dried sweet potato flakes. The combined application of oxygen absorbers and flexible oxygen barrier films resulted in significant retention of β-carotene in sweet potato flakes during 210 days of storage [139]. Furthermore, the red flour beetle showed a 100% mortality rate in packaged sultana raisins stored at 15–30 °C for 9–45 days. In the same study, the surface color was maintained by the application of an O_2_ absorber and storage temperature at 15 °C [140]. Oxygen absorbers hinder microorganisms in fruit and vegetable storage and additionally preserve the nutritional quality of food, such as vitamin concentration [137].

One of the crucial development steps in the fresh and minimally processed produce packaging sector is antimicrobial packaging systems, which are achieved by the incorporation of sachets or pads which contain volatile antimicrobial compounds, the application of antimicrobial and antioxidant packaging films and antimicrobial (edible or nonedible) coatings. Several alternative forms of antimicrobial compounds (chemical or natural, volatile or nonvolatile) may be either added into the packaging materials or directly applied onto the food surface with appropriate coating [141,142]. Three types of antimicrobial packaging have been reported for their applicability on fresh and minimally processed fruits and vegetable products, namely:

- Antimicrobial sachets: enclosure into packages of sachets that contain volatile antimicrobial agents
- Antimicrobial films: incorporation of volatile or nonvolatile antimicrobial compounds into the formulation of packaging films
- Antimicrobial edible coatings: direct application on the food surface of antimicrobial edible coatings or films. In this case, the main component of the coating may be a polymer with antimicrobial property (e.g., chitosan), or additional antimicrobial agents are added in the film-forming solution [6].

An antimicrobial sachet may be described as a pad that contains volatile antimicrobial agents, which is placed inside the food package to enable the gradual release of the antimicrobial and its interaction with the headspace in the package. The moisture concentration inside the package has been hypothesized as being the driving force for the release of the antimicrobial agent from the sachet system, and the released antimicrobial compounds inhibit the growth of microorganisms on the surface of the packaged fruits and vegetables [143]. Similar antimicrobial sachets containing alternative essential oils as antimicrobials have been reported as applicable for the inhibition of the growth of spoilage bacteria and pathogens in different products, including papaya, tomato and spinach leaves [69,70,71].

Antimicrobial film-forming materials are developed by incorporating the antimicrobial agents into a polymer matrix which allows their release onto the food surface to interact with the microflora. Zhang et al. [72] investigated the effect of a custom-made antimicrobial film by applying a coating containing sustained-release chlorine dioxide microcapsules on fresh mangos. According to a study reported by Ghaouth et al. [73], chitosan-coated tomatoes were adequately protected from *Penicillium* spp., *Aspergillus* spp., *Rhizopus stolonifer*, and *Botrytis cinerea*. Chitosan has been reported to provide appropriate control of fungal diseases, which may result in deterioration of fruit quality indices during storage [144]. Choulitoudi et al. [145] investigated the antioxidant effect of *Satureja thymbra* extracts, an ingredient rich in phenolic acids and flavonoids, after being sprayed on a non-edible, laminated packaging film for the prolongation of fried potato crisps’ shelf life. The tested indices were peroxide values, volatile products, and oxygen consumption. Moreover, the application of the extracts, either in active packaging or as additives onto the potato crisps, was investigated, with the former exhibiting the most significant antioxidant effect. Edible coating or film is referred to as a thin layer of material used for coating or wrapping several food products in order to decrease the rate of quality deterioration and prolong their shelf life. Edible films may be produced separately and subsequently applied to the foods, while coatings are formed directly onto the food surface [146]. In general, appropriate barriers to moisture and gases (mainly O_2_ and CO_2_ for fresh produce), uniform coating, adequate adhesion to food surface, colorlessness, and tastelessness are the basic requirements for an edible packaging system [147,148]. It is possible to apply coatings in various ways, including dipping, spraying, falling, or brushing. Quadros et al. [74] investigated the effectiveness of edible coatings with fish protein hydrolysate on the quality indices and shelf life of cherry tomatoes. The microbial growth results indicated that the protein hydrolysate-based edible coatings showed significant bioactivity as they inhibited the proliferation of molds and yeasts. Ruiz-Martinez et al. [75] reported the enhancement of the postharvest quality of tomatoes by an edible coating functionalized with a Flourensia cernua extract. Sortino et al. [76] evaluated the effectiveness of an edible coating based on Aloe arborescens and a combined treatment of 1-methylcycyclopropene and edible coating to improve the quality and prolong the shelf life of the “Settembrina” peach.

### 4.3. Smart Labels for Fruits and Vegetables

Smart packaging technology may provide crucial information regarding food quality, either indirectly (for example, Time Temperature Integrators) or directly (for example, freshness indicators) [149].

Freshness indicators may be referred to as appropriate devices that are incorporated into the sealed packaging of the food product and are intended to notify the end-user of the microbial and physicochemical changes that impact the quality level of the packaged food product. The interactions between the microbial metabolites and the indicators incorporated into the food package will visualize food quality in terms of microbial level [129,150]. Most of the proposed systems that have been developed up to now are based on the color changes of a label as a result of the microbial metabolism, which indicates that the packaged food may be no longer appropriate for consumption [151]. For fruits and vegetables, the state of freshness is commonly expressed within the context of ripeness. At a specific state of ripeness, it is often difficult to know when the fruit has entered the desired quality levels for the customer to use. For buyers, this condition often becomes an obstacle in understanding when the fruits should be purchased or consumed. The ripeSenseTM (www.ripesense.co.nz, accessed on 19 February 2021) has launched a ripeness sensor that enables the elimination of this problem. The sensor responds to the aromas that are released from the ripening fruit. The sensor is initially red and switches to orange and then yellow. This sensor has been used on pears and may also be used on other stone fruits, such as kiwifruit, melon, mango, and avocado [152]. Furthermore, the volatile compounds emitted during the different stages of fruit ripeness were analyzed using e-noses and used to assess the ripeness of tomatoes [153]. A color indicator based on bromophenol blue placed on the food package has been designed, and preliminary tests have been carried out to evaluate the freshness of guava (*Psidium guajava* L.) samples by [154]. A freshness sensor has been developed by Kemiklioglu and Ozen [119], which works with the principle of measurement of ion concentration change occurring in fruits and vegetables.

A Time Temperature Integrator (TTI) is defined as an inexpensive, smart label that may show as easily measurable time- and temperature-dependent change, reflecting the time-temperature history of the product to which it has been attached [155]. The principle of TTI operation is a mechanical, chemical, electrochemical, enzymatic or microbiological irreversible change, which is mainly expressed as a visual response (e.g., mechanical deformation, color change, or movement). During the last thirty years, several alternative TTI systems have been developed; however, a limited number have reached the industrial prototype stage, and even fewer have found a commercial application [125]. The chronology of the development of TTI has been summarized by Taoukis [156]. TTIs have been reported as effective tools for quality and shelf-life monitoring of perishable foods, mainly meat and fish products, in the cold chain. For these food product categories, temperature plays a critical role in quality degradation and shelf life at any stage of the supply chain. In Bobelyn et al. [157], a feasibility study has been described with the aim to evaluate the potential of TTI application as indicators of the quality of horticultural products. In this study, mushroom (Agaricus bisporus Pilát) has been used as a case study. An appropriate enzymatic TTI (Vitsab A.B., Malmö, Sweden) has been used for quality monitoring of mushrooms at constant and variable temperature conditions.

## 5. Conclusions

The demand for fresh, nutritious, and convenient food by consumers has recently shown a constant rise. New product development and the diversification of fruit- and vegetable-based food products have supported this trend. Whilst addressing the consumer demand for novel, nutritious, and affordable products, food producers must consider also the requirement for food safety assurance and shelf-life extension for the developed products. A scientific challenge for the food sector is to investigate the potential of shelf-life extension and the development of appropriate shelf-life predictive models [67]. In general, minimal processing has shown the potential to further extend the shelf life of fruits and vegetables by the combined application of different preservative hurdles, as for example low temperature, natural or synthetic antimicrobials and antioxidants, a_w_, pH, and high pressure with packaging methods, such as modified atmospheres and active packaging. Future research should be focused on the development of mild processing techniques, in the context of hurdle technology, that retain the fresh-like characteristics while at the same time extend the shelf life of the final products without jeopardizing their safety and integrity. Additionally, the introduction of optimal active and smart packaging methods by the incorporation of alternative active compounds or active and smart functionalities in a new, combined system may also contribute to this objective.

## Figures and Tables

**Table 1:** Indicative studies on the application of hurdle technology on fresh-cut fruits and vegetables (Abbreviations: RPL: repetitive pulsed light; OD: osmotic dehydration; HHP: high hydrostatic pressure; SAEW: slightly acidic electrolyzed water; US: ultrasound; MH: mild heat treatment; AA: ascorbic acid; HR: 4-hexylresorcinol; CM: chamomile; MAP: modified atmosphere packaging; PP: polypropylene; GO: garlic oil; AIT: allyl isothiocyanate; TVC: total viable count; PME: pectin methylesterase; PG: polygalacturonase; TAA: total antiradical activity).

Matrix Tested	Hurdles Applied	Main Indices Studied	Main Results	References
Fresh-cut cantaloupe	Edible (alginate) coating Packed into polypropylene bag Repetitive pulsed light (RPL) Chill storage	aerobic mesophilic plate count, anaerobic mesophilic plate count and yeast and mold populations	A 28- and 24-day shelf-life extension of the combined method compared to the untreated and alginate-coated samples, respectively.	[54]
Fresh-cut tomatoes	Osmotic dehydration (OD) in concentrated solutions (glycerol (50% *w*/*w*), erythritol (12.5% *w*/*w*), sodium chloride (3.5% *w*/*w*), calcium chloride (1.5% *w*/*w*) and Citrox (0.2% *w*/*w*) at a temperature of 35 °C for 90 min) High Hydrostatic pressure (HHP) (600 MPa −25 °C for 5 min) Chill storage	Total aerobic viable count (TVC) and yeasts and moulds PME, PG Lycopene Color Sensory evaluation	A 74- and over 240-day shelf-life extension of OD and the combined method (OD-HHP) compared to the untreated samples, respectively.	[55]
Fresh-cut apple	Washing with chlorinated water Dipping in ascorbic acid/calcium chloride solution Edible coating (pectin powder + apple fiber) Placed into transparent polypropylene trays, sealed, with a 64 μm thick polypropylene film PL treatments	Antioxidant capacity (DPPH method) Color/firmness measurement Mesophilic and psychrophilic aerobic microorganisms, yeasts, and molds Sensory acceptability	The combination of Pectin coating and PL resulted in an almost 2 log CFU g^−1^ reduction of microbial counts, and apple pieces exhibited higher antioxidant activity values. Sensory attribute scores did not fall below the rejection limit during 14 days.	[56]
Fresh-cut bell peppers	Slightly acidic electrolyzed water (SAEW) Ultrasound (US) Mild heat treatment (MH) at 45, 50, and 60 °C	*Listeria monocytogenes* and *Salmonella enterica* serovar *Typhimurium* Texture and color analysis	The optimized condition of the combined treatment was SAEW + US + 60 °C for 1 min, with minimal changes in quality indices	[57]
Fresh-cut apple	Ultrasound Ascorbic acid	Enzyme inactivation (monophenolase, diphenolase, and peroxidase)	The combined application of ultrasound and ascorbic acid showed synergistic inhibitory effects on enzymes related to enzymatic browning.	[58]
Fresh-cut peach and apricot	Osmotic dehydration (OD) in concentrated solutions (glycerol (50% *w*/*w*), erythritol (12.5% *w*/*w*), sodium chloride (3.5% *w*/*w*), calcium chloride (1.5% *w*/*w*) and Citrox (0.2% *w*/*w*) at a temperature of 45 °C for 45 min) High Hydrostatic pressure (HHP) (600 MPa −25 °C for 5 min) Chill storage	Total aerobic viable count (TVC) and yeasts and moulds Color Sensory evaluation Texture	Shelf-life estimated at 309 and 320 days at 4 °C, for OD/HP peach and apricot, respectively, compared to approximately 68–86 days, for OD samples, when non-treated samples are expected with a shelf life of 5–7 days at cold storage.	[59]
Fresh-cut apples	carboxymethyl cellulose (CMC)and aloe vera coatings antibrowning agents (ascorbic acid (AA), CaCl_2_, cysteine, 4-hexylresorcinol (HR)) Samples packaged in polypropylene containers, shrink-wrapped, and stored at 5 ± 2 °C	Ascorbic acid, total phenols, and antioxidant activity Polyphenol oxidase and peroxidase activity Sensory evaluation Microbial enumeration Browning and whiteness index Weight loss and fruit firmness	Combined application of CMC (1%) with HR (0.01%) + AA (0.5%) + CaCl2 (0.2%)—the most effective treatment for quality preservation and reduction of surface browning in fresh-cut wedges during storage for 7 days at 5 ± 2 °C.	[60]
Fresh-cut lotus roots	antibrowning agents (ascorbic acid (AA), chamomile (CM)) heat treatment (55 °C for 45 s) MAP packaging (air, vacuum, 100% CO_2_, 50% CO_2_/50% N_2_)	color weight loss texture pH polyphenoloxidase activity total phenolic content	Dipping in an AA + CM antibrowning treatment and 100% CO_2_ MAP with a heat treatment extended the shelf-life to 21 days at 5 °C.	[61]
Fresh-cut apples	antibrowning agents (ascorbic 1 citric acid; ascorbic acid 1 NaCl; NaCl, citric acid, and Ca-ascorbate) Ultrasound (1 and 3 min)	Determination of soluble solids content and pH Color Sensory evaluation	The most effective antibrowning solution was Ca-ascorbate. Longer application of US may be beneficial for the prevention of browning in fresh-cut apples.	[62]
Fresh-cut strawberry	Osmotic dehydration (OD) in concentrated solutions (glycerol (50% *w*/*w*), erythritol (12.5% *w*/*w*), ascorbic acid (2.0% *w*/*w*), calcium chloride (1.5% *w*/*w*), citric acid 1.0% *w*/*w*), Citrox (0.1% *w*/*w*) and L-cysteine HCl (0.20% *w*/*w*) at 15, 25, 35, and 45 °C for times up to 300 min High Hydrostatic pressure (HHP) (600 MPa −25 °C for 5 min) Chill storage	Total aerobic viable count (TVC) and yeasts and moulds Color Sensory evaluation Total antiradical activity (TAA) Identification of anthocyanins Texture	Shelf life was significantly extended for both OHP and OD (up to 10 and 4 months at 5 °C, respectively) compared to untreated samples (7 days at 5 °C). The addition of L-cysteine added in the OD solution exhibited an exceptional red color intensity and stability.	[63]
Fresh-cut rocket	UV-C radiation (5, 10, and 20 kJ/m^2^) gaseous ozone (1, 2, and 5 ppm for 10 min) placed in polypropylene (PP) trays were sealed at the top with a bioriented PP film of 35 μm (passive MAP) cold storage	Chlorophyll and Carotenoid Content Color Sensory evaluation Enumeration of mesophilic, psychrotrophic and enterobacteria, molds, and yeasts	The 20 kJ UV-C/m^2^ treatment reduced the microbial load of the fresh-cut rocket during 8 days of storage at 5 °C.	[64]
Freshcut pear cubes	Blanching at 95 °C for 3 min in a 17 °Brix aqueous solution containing High Fructose Corn Syrup and citric acid MAP (N_2_/CO_2_ (80:20 *v*/*v*)) cold storage	Color Drip loss Firmness Sensory evaluation Total aerobic population, yeasts and molds, Pseudomonadaceae, and total coliforms	The combination of mild heat treatment (3 min at 95 °C)/MAP under aseptic conditions improved the stability.	[65]
Fresh-cut leafy salad (lollo rosso lettuce, lollo verde lettuce, and rocket)	Low temperature MAP (3% O_2_, 10% CO_2_, 87% N_2_)	Total viable count, *Pseudomonas* spp., lactic acid bacteria, vitamin C, color, and texture	Development and validation of adequate predictive shelf-life models.	[66]
Dandelion leaves	Low temperature MAP (22% CO_2_–78% N_2_)	Total viable count, *Pseudomonas* spp., lactobacilli, yeasts, and molds, *Enterobacteriaceae* spp., texture, enzymatic activity, vitamin C concentration and sensory evaluation	MAP resulted in a 1-day extension of shelf life compared to conventional aerobic storage in perforated films. Development and validation of adequate predictive shelf-life models.	[67]
Plum and strawberry	Low temperature Modified atmosphere and humidity packaging	Moisture loss, gas and water vapor transfer	Development and validation of adequate simulation program for the preservation of packed fruit.	[13]
Peach and cherry tomatoes	Low temperature Equilibrium modified atmosphere packaging (1–3% CO_2_, 85% RH)	In package CO_2_, O_2_, and C_2_H_4_	Biodegradable laser-microperforated PLA films were designed for fruit packaging and preservation.	[68]
Fresh-cut tomatoes	Low temperature Active pads with encapsulated garlic oil (GO)	GO volatile release, total viable count, yeasts and molds, coliforms and *E. coli*, sensory evaluation	Tomato was affected by the highest concentration of GO capsules, showing lower microbial growth and higher sensory quality.	[69]
Papaya	Antimicrobial sachet containing oregano, cinnamon, and lemongrass EO	Antifungal activity, weight loss, peel colour and firmness, total soluble solids, titratable acidity	Sachets containing cinnamon, oregano, and lemongrass resulted in a significant reduction in the growth of mesophilic aerobic bacteria, yeasts, and molds.	[70]
Fresh spinach	Low temperature Antimicrobial sachet containing allyl isothiocyanate (AIT) vapor	AIT release, antimicrobial effect against *E. coli* O1§75:H7, yeasts, and moulds	The load of *E. coli* O157:H7 on spinach leaves decreased by 1.6–2.6 log CFU/leaf at 4 °C and 2.1–5.7 log CFU/leaf at 25 °C within 5 days.	[71]
Mangoes	Low temperature Antimicrobial film coated with chlorine dioxide microcapsules	Weight loss, firmness, colour, soluble solids, vitamin C, titratable acid, and nutritional value	Mango showed quality degradation after 21 days of storage. The chlorine dioxide microcapsule antibacterial film preserved the high quality for longer storage periods.	[72]
Tomatoes	Low temperature Chitosan coating	Respiration rate, ethylene	Coating increased the internal CO_2_ concentration and decreased the internal O_2_ levels in tomato samples.	[73]
Cherry tomatoes	Low temperature Edible coatings	Color, weight loss, firmness, soluble solids, pH, and molds and yeasts	The edible coating with protein hydrolysate inhibited the proliferation of molds and yeasts.	[74]
Tomatoes	Edible coating with *Flourensia cernua* extract	Sensory evaluation, weight loss, firmness, pH, colour	The edible coating incorporated with F. cernua extract was the most effective in delaying pathogenic fungi growth and preserving the visual appearance of the final product at the end of the storage period.	[75]
Peach	Low temperature Edible coating containing 1-Methylcyclopropene and Aloe arborescens	Carotenoids content, phenolic content, reducing activity, titratable acidity, total soluble content, weight loss, and vitamin C content	The single and combined application of aloe-based coating slowed down the maturation processes of the fruit, delayed the weight loss, and preserved the sensory properties of the final products.	[76]
